# From political priority to service delivery: complexities to real-life priority of abortion services in Ethiopia

**DOI:** 10.1093/heapol/czae061

**Published:** 2024-07-08

**Authors:** Emily McLean, Ingrid Miljeteig, Astrid Blystad, Alemnesh H Mirkuzie, Marte E. S Haaland

**Affiliations:** Bergen Center for Ethics and Priority Setting, Department of Global Public Health and Primary Care, University of Bergen, Årstadveien 21, Bergen 5020, Norway; Medical department, Nordland Hospital, Ivar Bergsmoes gate 3, Stokmarknes 8450, Norway; Bergen Center for Ethics and Priority Setting, Department of Global Public Health and Primary Care, University of Bergen, Årstadveien 21, Bergen 5020, Norway; Global Health Anthropology Research Group, Department of Global Public Health and Primary Care, University of Bergen, Årstadveien 21, Bergen 5020, Norway; John Snow Research and Training Inc, Edna Mall Area, Addis Ababa, Ethiopia; Institute for Health Metrics and Evaluation, University of Washington, 3980 15th Ave NE, Seattle, WA 98195, USA; Global Health Anthropology Research Group, Department of Global Public Health and Primary Care, University of Bergen, Årstadveien 21, Bergen 5020, Norway

**Keywords:** Abortion, priority-setting, Ethiopia, health policy, policy implementation, reproductive health, global health, maternal health

## Abstract

Improving access to abortion services has been coined a high priority by the Ethiopian Federal Ministry of Health. Nevertheless, many women are still struggling to access abortion services. The dedicated commitment to expanding abortion services by central authorities and the difficulties in further improving access to the services make for an interesting case to explore the real-life complexities of health priority setting. This article thus explores what it means to make abortion services a priority by drawing on in-depth interviews with healthcare bureaucrats and key stakeholders working closely with abortion service policy and implementation. Data were collected from February to April 2022. Health bureaucrats from 9 of the 12 regional states in Ethiopia and the Federal Ministry of Health were interviewed in addition to key stakeholders from professional organizations and NGOs. The study found that political will and priority to abortion services by central authorities were not necessarily enough to ensure access to the service across the health sector. At the regional and local level, there were considerable challenges with a lack of funding, equipment and human resources for implementing and expanding access to abortion services. The inadequacy of indicators and reporting systems hindered accountability and made it difficult to give priority to abortion services among the series of health programmes and priorities that local health authorities had to implement. The situation was further challenged by the contested nature of the abortion issue itself, both in the general population, but also amongst health bureaucrats and hospital leaders. This study casts a light on the complex and entangled processes of turning national-level priorities into on-the-ground practice and highlights the real-life challenges of setting and implementing health priorities.

Key messages

- Even though abortion services had been coined a high priority by Ethiopian Federal Ministry of Health, maintaining political priority towards the service as it travelled through the health sector was challenging.
- Lack of funds created a dissonance between priority given to the service by federal authorities and the possibility to implement the service by regional authorities.
- Inadequacy of indicators and reporting systems reduced accountability towards abortion services, making it harder to maintain priority towards the service amongst other pressing health programs
- Abortion is a contested issue in the general population, amongst health bureaucrats and hospital leaders. This further challenged the priority and hence implementation of abortion services.

## Introduction

‘We prioritize safe abortion’ was proudly stated by a federal health bureaucrat as she explained the increased availability of abortion services in Ethiopia and the achievements made in reducing deaths from unsafe abortion. Her colleague went on to explain that abortion services were now a high priority for the Ministry of Health and that; ‘we are living witnesses to how safe abortion services have transformed the maternal and child health services in the country’.

A dedicated commitment to reducing unsafe abortions is an important pre-condition for giving priority to the implementation of abortion services. As such, Ethiopia constitutes an interesting case to explore opportunities and challenges when translating priority in terms of political will to the distribution of budget funds and actual implementation throughout the healthcare system. In this article we raise the question; what does it mean to make abortion a health priority?

Ethiopia has been recognized for introducing a relatively liberal legal framework for abortion (Center for Reproductive Rights, 2024). In 2005, changes were made to the criminal code to decriminalize abortions in cases of rape or incest, if the pregnant woman is a minor or has disabilities, to save her life or in cases of foetal malformation (FDRE, 2005). Such conditions required for accessing safe abortion services are common in abortion legislation, but the Ethiopian guidelines that were developed alongside the legal reform state that the abortion-seeking woman’s word should be sufficient to prove that she fulfils the criteria, without the need for proof or further investigation (Wada, 2008; FDRE, FMoH, 2014). These changes have granted access to legal and safe abortions to a large number of Ethiopian women (Gebrehiwot *et al*., 2016; Moore *et al*., 2016).

The change in the criminal code in Ethiopia came about as a result of political will to address daunting numbers of maternal mortality due to unsafe abortions (Wada, 2008; Bridgman-Packer and Kidanemariam, 2018; Blystad *et al*., 2019; Holcombe and Gebru, 2022). As demonstrated by the opening vignette to this article, there is reason to believe that this will is still present among central decision-makers in Ethiopia. This sets Ethiopia apart from many countries in the region, where the political will to liberalize abortion services has been limited (Blystad *et al*., 2019; Haaland *et al*., 2020; Suh, 2020).

Healthcare calls for tough priorities between much-needed health services (Ham, 1997; Daniels and Sabin, 2008). This is particularly so in low- and middle-income countries where budget constraints are considerable (Glassman *et al*., 2012; Hipgrave *et al*., 2014). In 2019, the Ethiopian government spent 36 dollars per person on health (Partnership and Cooperation Directorate, FMoH, 2022). In comparison, an average high-income country spends 5938 dollars per person (Partnership and Cooperation Directorate, FMoH, 2022; Micah *et al*., 2023). With a broad range of health needs among Ethiopia’s approximately 107 million inhabitants, setting the right health priorities is challenging (Institute for Health Metrics and Evaluation, 2023).

In 2019, the Ethiopian Federal Ministry of Health (FMoH) launched the second part of its ten-year Health Sector Transformation Plan (HSTP-II) detailing areas of priority for the public healthcare sector for the coming 5 years (FMoH, 2021; Eregata *et al*., 2020). Abortion was stated as a high priority, with the aim to expand access to and improve the quality of the service across the country (FMoH Ethiopia, 2019).

Abortion remains a complex issue in Ethiopia. Despite the change of law and stated priority given to abortion services, women still struggle to access such services, particularly in rural areas (Sheehy *et al*., 2021; Feyssa and Gebru, 2022). Several regions do not meet the requirements for abortion service availability, and even in the regions that do, women often need to travel far to reach the service (Dibaba *et al*., 2017). Estimates indicate that 47% of abortions occur outside of health facilities, making them potentially unsafe (Moore *et al*., 2016). The majority of women do not know about their right to abortion (Geleto and Markos, 2015; Sheehy *et al*., 2021; Zimmerman *et al*., 2022). Abortion-related stigma and disrespectful treatment of abortion-seeking women are documented and abortion providers face discrimination (McLean *et al*., 2019; Bercu *et al*., 2022). There is moreover a lack of health workers willing to offer the service, and stock-outs of abortion drugs and equipment are not uncommon (Feyssa and Gebru, 2022). More than 15 years after the legal reform, abortion remains a controversial topic in Ethiopia, and it is rarely spoken about in public (Blystad *et al*., 2019; Tadele *et al*., 2019). Though little information is available on anti-abortion groups in Ethiopia, there is a growing concern amongst key stakeholders working closely with abortion that these groups are on the rise (Hellerstein, 2023). The Ethiopian abortion service provision thus constitutes an example where there seems to be political will, legal ground and priority given to abortion, but simultaneously there are considerable obstacles to further expanding access to abortion services.

In this article, we will assess how the gap between political priority to abortion, and the challenges to further expand access to abortion services, needs to be understood in terms of how priorities are communicated and implemented across the healthcare system. Hipgrave *et al*. (2014) have highlighted this challenge in their study stating that we need a better understanding of how priorities are linked between the different levels of the healthcare system. Whilst there has been important research in the priority-setting literature on frameworks, criteria and processes for setting fair health priorities, less attention has been given to the real-life implementation and understanding of health priorities across the healthcare sector, especially in low- and middle-income countries (LIMICs) (Kapiriri and Martin, 2006; Daniels and Sabin, 2008; Hipgrave *et al*., 2014; Cromwell *et al*., 2015; Essue and Kapiriri, 2020; Seixas *et al*., 2021). Researchers have expressed a wish for better integration of contextual aspects like health infrastructure, power dynamics and local politics into our understanding of priority-setting (Hipgrave *et al*., 2014; Kapiriri and Razavi, 2017). What happens after a health service has been set as a priority? And what influences its implementation across the health sector?

To understand why political will and priority by central authorities do not seem to necessarily ensure access to abortion services, there is a need to explore the processes through which such political will and overall priority setting are translated into services implementation. This article explores the practical implications of priority setting by examining the case of the Ethiopian abortion policy. It aims to contribute to filling a knowledge gap on the processes through which national priorities for health are translated into actual services. Drawing on discussion with key stakeholders, we will explore the processes through which health system priorities are implemented and translated across the health sector. The article aims to contribute to a better understanding of the articulation between priority setting at the policy level and at the level of service provision.

## Materials and methods

This study employed a qualitative approach based on in-depth interviews with healthcare bureaucrats and practitioners working closely on abortion service policy and implementation. Data were collected from February to April 2022.

### Study setting

Ethiopia is the second most populated country on the African continent with a population of approximately 107 million (Institute for Health Metrics and Evaluation, 2023). It is a federal republic comprised of 11 regional states (Tigray, Afar, Amhara, Oromia, Somali, Bensihangul-Gumuz, SNNPR, Gambella, Harari, South Western and Sidama) and 2 city administrations (Addis Ababa and Dire Dawa). In this study, regions also refer to the two city administrations. The regions are further divided into woredas (districts). Ethiopia has a decentralized governance structure in which regions have the right to self-governance (Gebremariam and Kidanemariam, 2023). The national abortion law is binding for all regions.

The health sector is organized through the federal, regional and district levels. The Federal Ministry of Health (FMoH) functions as an advisor, developing policy, guidelines and standards for health care for the entire country. FMoH also runs tertiary-level hospitals and distributes financial and medical resources. At the regional level are the Regional Health Bureaus (RHB). They are implementors through the running of general and primary hospitals and specialized health centres. They also decide how un-earmarked funding is spent within their region. At the woreda level are the District Health Offices (DHOs). The DHOs oversee local health centres, health posts and health extension workers.

Funding of the Ethiopian health sector is fragmented, and the many differently positioned actors involved make the process challenging (Alebachew, A; Yusuf, Y; Mann, C; Berman, P.; FMoH, 2015). There are three main channels of funding in addition to finances generated through out-of-pocket expenditure: (1) Funding from the federal government to the regional government as both earmarked and non-earmarked funding. The regional government then distributes these funds to their regional departments under which RHB is one. (2) Funding, drugs and equipment from donors channelled through FMoH, which further distributes it to RHBs. (3) Donor money allocated directly to specific projects, health facilities or RHBs outside the government system (Alebachew, A; Yusuf, Y; Mann, C; Berman, P.; FMoH, 2015).

Studies show that the amounts of funding allocated for health at the regional level vary from 6% to 13% of the total regional expenditure (World Bank Group, 2016). The amount ending up at each health facility is dictated by need and population size; 34% of funds for health in Ethiopia come from donors, followed by 30% from out-of-pocket payments, 21% from the regions and woredas and 10% from the federal government (Partnership and Cooperation Directorate, FMoH, 2022).

The latest estimate on the abortion rate in Ethiopia is from 2014 and showed a rate of 28 abortions per 1000 women aged 15–49 years, with Addis Ababa having the highest rate at 92 per 1000 (Moore *et al*., 2016). Abortion is provided at public, private and NGO facilities, with the majority of women (66%) seeking care from private clinics and NGOs (Moore *et al*., 2016). Both manual vacuum aspiration (MVA) and medical abortion (MA) are used. Data on the use of these methods are limited to a study from 2014 showing that MVA accounted for 52.7% of abortions, and MA for 35.6% (Gebrehiwot *et al*., 2016). A small study from Addis Ababa in 2018 showed that most women used medical abortion (Yeshambel Wassie *et al*., 2021).

In March 2020, the first case of Covid-19 was reported in Ethiopia (World Health Organization, 2024). Ethiopia experienced a low case fatality rate from the pandemic compared with other African countries, and although there were economic losses at the start of the pandemic, the economy quickly bounced back to almost pre-pandemic levels (Harris *et al*., 2021). Studies show that sexual and reproductive health services were negatively affected during the initial phase of the pandemic with a reported shortage of abortion drugs and challenges with accessing safe abortion care (The African Population and Health Research Center, IPAS Africa Alliance, Amref Health Africa *et al*., 2021; Feyssa and Gebru, 2022). In addition, Ethiopia experienced an internal conflict in the northern regions of the country starting in November 2021. The conflict left thousands of people dead and internally displaced and caused great damage to infrastructure, including health institutions (Center for Preventive Action, 2023). Though the conflict was in regress at the time of data collection, it is believed that it had an important impact on government budgets and hence health priorities and sexual and reproductive health services, as resources were rerouted towards the conflict (Feyssa and Gebru, 2022).

### Study participants

To increase the understanding of how abortion is prioritized and implemented at different levels of the public health sector, the study employed a strategy of purposive sampling. With this approach, we aimed to recruit health bureaucrats who were directly involved in working with abortion service policy and implementation at the federal and regional level, and personnel employed in NGOs and members of professional organizations working with abortion services.

Study participants were recruited by the first author with assistance from the Ethiopian Public Health Institute. With the complexity of culture, norms and geography in Ethiopia, we recruited participants from different regions to gain a diversity of viewpoints. Potential participants were identified during the initial phase of the fieldwork. As the study progressed, more participants were identified through snowballing. Recruitment was conducted via email or text message.

In total, the study included 23 participants. Of these, 11 were regional health bureaucrats working with abortion services at 9 of the 12 RHBs in Ethiopia. Health bureaucrats from the remaining three regions declined to participate in the study. Two participants were federal health bureaucrats working with abortion at the FMoH. To gain a wider perspective, personnel employed in NGOs and members of professional organizations working with abortion services were also invited to participate in the study. Of these, three were members of professional organizations and seven were personnel from NGOs.

### Data collection

Data were collected through semi-structured interviews conducted by the first author in English. The interviews lasted between 30 and 55 minutes. The first author carried out the interviews either in-person in Addis Ababa or via Zoom with participants in other regions due to the ongoing security issues which made it difficult to travel outside the capital. General questions about the abortion law and services and more in-depth questions about challenges and dilemmas related to abortion policy, implementation and priority were asked. The interview guide was constantly revised during the fieldwork to accommodate relevant emerging themes. New participants were recruited until no major new themes were brought up by the participants. All the interviews were audio-recorded and transcribed verbatim by the first author and a professional transcription company.

### Analysis

This study has drawn upon thematic analysis (Attride-Stirling, 2001). The analysis followed an iterative approach, shifting between the participants’ perspective and the overarching context they worked within. While the interviews were coded inductively, the analysis process was overall informed by the ongoing debates about priority settings in low- and middle-income countries. The first author initiated the analysis process during fieldwork, noting new themes of interest as they emerged in the interviews. After data collection was completed, the first author read all the interviews to gain an overview of the full material and major themes emerging. A sample of the transcripts was read by all authors to gain familiarity with the collected material. The first author ascribed codes to organize the material and further grouped it into common themes (Attride-Stirling, 2001). Themes and codes were discussed with the rest of the co-authors before the themes were adjusted and revised, and interviews were re-read to check for consistency (Attride-Stirling, 2001). Nvivo12 software was used in the coding of the material (QSR International Pty Ltd, 2020).

### Ethics

Ethical approval for the study was granted by the Ethiopian Public Health Institute Institutional Review Board (ID-no: EPHI-IRB-402-2021) and the Regional Ethical Committee in Norway (ID-no: 249828). The study participants were informed about the objective of the study, the research’s ethical principles of voluntary participation, confidentiality, the possibility to withdraw at any given time and the right to not answer questions. They were also informed about the purpose of the study. Personal information was kept in a secure place. No compensation was provided for participation. Particular care was taken to protect the anonymity of the participants. For this reason, the regions where the participants worked are not mentioned by name and personal details in the quotes that might reveal the identity of the participants have been removed. All participants signed a consent form to enter the study.

## Results

### ‘There is no special budget allocated’

Even though federal health authorities emphasized that abortion is placed high on the priority agenda, several of the study participants expressed concern about the willingness and possibility to implement abortion services; *‘*we have a plan to expand safe abortion services in the government health facilities, but that needs resources, and we have some constraints’. Limited financial resources to enable implementation was a recurrent issue in our interviews. A regional health bureaucrat explained, ‘I have been trying to talk with the head of the health bureau to allocate even a minimal amount of money to cover the cost of comprehensive abortion care, but they said that there are budget shortages, so it is difficult to allocate money to this’.

Limited funding for health services was not a new problem in the public healthcare sector. Yet many participants expressed a feeling that other health services were receiving more financial resources than abortion services; ‘there is no special budget allocated [for abortion services] by the government because there are other priorities like pregnancy, deliveries, and post-natal care. These are the priorities of the government’. This often led to frustration among the study participants, since abortion services were not given the appropriate attention and financial resources that were expected with it being a prioritized service.

There was often a lack of trained health workers as a regional health bureaucrat explained that ‘the health workers may leave and then the health centre is without a trained health worker. For most health centres the training is very expensive and takes a lot of the budget’. Stock-out of essential abortion drugs and equipment was not uncommon. Health workers trained in abortion care were in some health facilities reallocated to other tasks, leaving nobody to care for abortion patients. In some regions, these shortcomings meant that few public health facilities were able to provide abortion services, as another regional health bureaucrat explained, ‘almost 50% of public health facilities are not providing abortion services in our region’.

Most regions were relying on external funding and clinics run by NGOs and donors to cover the cost of abortion service provision. In some urban regions, this system had worked well for years, whilst most rural regions were having difficulties attracting external funders. The system was moreover fragile and vulnerable to sudden change, as elicited by a regional health bureaucrat; *‘*we are struggling as most of the health services were financed by partners, but you may know the current situation in our country [the internal conflict], with this lots of partners were lost and still the domestic budget is not allocated for specific programs like abortion care’. The covid-19 pandemic had also affected the availability of funding further worsening the financial situation for abortion services.

Many participants reported a feeling of dissonance between their expectation of financial resources given that abortion was a stated priority and the reality of scarce funding hampering implementation. One participant stated that ‘safe abortion services are considered [by the regional and federal government] to be an NGO activity, this is the understanding in our system. There is no financing from the federal or local government’. Another added, ‘There are priority programs that the government [federal and regional] is giving attention to and this [abortion] is just considered secondary’.

### ‘They do not prioritize abortion as the other health programs’

The federal and decentralized structure of Ethiopia leaves responsibility for implementing health services to the regional governments. Such a decentralized system creates a situation where priorities need to be set by all levels of administration to take effect. Participants expressed concerns about what happened as the abortion priority travelled from the federal to the regional, district and eventually to the health facility level. Many believed that priority towards abortion services faded along the way. When bureaucrats, from time to time, supervised health facilities, they often found that rooms meant for abortion services were repurposed for other health services or that there was a lack of a dedicated space in the health facilities to provide abortion services. As a regional health bureaucrat explained;

Although we have a favourable environment for abortion care […] we still have difficulties at the lower level. When you go to the districts, there’s a frequent change of health leaders and sometimes they don’t own the program and they do not prioritize this as the other programs. For example, if it is maternal health or delivery, they’re more responsible because it is a key performance indicator for them. Or it could be the HIV program, they cannot perform low because they will be held accountable.

The regional health bureaucrat not only raises the challenges of maintaining abortion services as a priority in lower levels of health administration but also points to the structure of incentives favouring other health services over abortion. The lack of attention and accountability mechanisms for abortion services by all levels of government was affecting the actual level of priority it received. Although data on the number of women receiving abortion services in the public sector exist, this information was not openly available to the public and it was alluded by a few of the study participants that these data were insufficient and had errors or shortcomings, ‘abortion, as you know, it is stigmatized wherever you go, and we don´t have adequate evidence or data on abortion compared to the other reproductive health issues’. Another added that ‘some surveys show that still now unsafe abortion is contributing nearly 6-10 per cent of maternal death, but our routine tool does not capture that number’.

Participants reported that key indicators to measure abortion provision, quality and progress were either entirely missing or lacking in quality, as other health issues were considered more important to register,

When we see the indicators by the Ministry of Health there is only a single indicator which measures the access of comprehensive safe abortion care services [the number of safe abortions performed] it does not measure the safe abortion service provisions, it doesn´t measure the service integration with family planning and it does not measure the awareness or the concern of the value of health care providers.

The inadequacy of indicators also affected to what degree abortion services were being monitored and evaluated. Although this varied across the regions, and one region was firm in stating that they did conduct routine monitoring of their services, this was not the case in all regions:

They [the health program implementors at the regional level] give less attention to the [abortion] services. They don’t monitor nor evaluate the abortion service. If a service has not been monitored and evaluated continuously, the improvement and progress of the service will be weak. Then we cannot obtain the final objective or goal. The main health system obstacle is owning the safe abortion program as the main health program, as a health service that we track. Due to that, the service providers also give less attention to the safe abortion service.

Insufficient abortion-related indicators, limited systems of reporting and absence of monitoring raised questions among participants about the actual level of priority given to abortion by the national and regional governments.

### ‘Abortion care service is considered a sin’

While all the study participants expressed their support for public abortion services, they also pointed to persistent negative attitudes towards abortion in society at large, especially so in rural areas. Abortion was seen to be sensitive and morally questionable as a regional health bureaucrat explained; ‘giving abortion services is considered a sin’ and another added, ‘You cannot just talk about it [abortion] in front of others, because they are seeing it as a sin, as unsafe sex and abortion are forbidden’. There was a growing concern among participants that these views were indeed on the rise in Ethiopia, as anti-abortion groups had become more vocal and present in the public sphere during the Covid-19 pandemic, ‘they [anti-abortion groups] are talking a lot about abortions, especially associating it with Covid, telling people that Covid is due to the abortion service we are doing here’. Such sentiments added to the stigma and taboo associated with abortion, further challenging the implementation and expansion of abortion services.

Participants described how negative attitudes towards abortion affected the recruitment of health workers to provide abortion services, as a regional health bureaucrat explained; ‘giving abortion care service is considered a sin, that is why most health workers leave’. Bureaucrats similarly talked about a high turnover and lack of abortion providers. Although high turnover was commonly seen across the public health sector as health workers tended to move onto better-paid jobs or move to urban areas, it was believed that the high stigma associated with providing abortion services was a strong driving factor, ‘we train them and when they return back to their facility and after a certain time serving, they decide that they do not want to perform such a service because of their religion’. Some participants described cases of serious stigma towards abortion providers, including incidents where abortion providers were named at church services, creating a fear of continuing to provide the services. Amongst hospital managers abortion was also sensitive, sometimes leading to a lack of support for the implementation of the service; ‘even the medical directors you see. For example, if you want to implement [the abortion service], you have to fight a lot with the medical directors’.

Even though abortion was a stated priority by central authorities, negative attitudes towards abortion were also encountered within the health bureaucracy. One study participant reflected, ‘I think about the abortion law in Ethiopia, it’s very much liberal and accommodative, it’s one of the best in Africa. But the problem is, despite the presence of this very good legislation, the officials, even at the ministry level, at all levels of the system, are quite restrictive’. Another added, ‘Still you find people from the government having personal opinions about abortion which can become an obstacle. They are not pro-abortion, not supporting women having access to abortion’. Some feared that these sceptics found within the bureaucracy led to reduced attention and concern for the priority and provisions of abortion services, ‘This [the value issue] may be the reason why the higher officials and program owners are [not paying attention to the service]’.

Throughout the interviews, it became clear that considerable care had to be taken when bureaucrats raised issues related to abortion within their government structures. Several study participants explained that they had been told by higher-level government officials to keep a low profile when talking about abortion, ‘They don’t want to come out bold and push the issue forward. The policy is very progressive and good, but they want us to be silent’. In some regions, bureaucrats could not talk openly about abortion at all due to the political sensitivity of the issue, and several NGOs even refrained from working in these regions due to the unfavourable political and administrative environment. The degree of negative sentiments towards abortion seemed to vary substantially between the different regions.

Negative attitudes were sometimes linked to a lack of awareness about abortion-related complications and the implications of lacking accessibility of the services, ‘our higher and lower-level officials have a shortage of awareness about the consequences of limiting the service’. Without sufficient knowledge of the possible consequences of not offering safe abortion services, bureaucrats were left with few incentives to prioritize abortion services over other pressing health needs. Elaborating on this point, a study participant reflected on how the narrative about the decrease in abortion-related deaths over the past few years had led to less concern about the abortion issue, reducing the need to prioritize abortion services: ‘It used to be a priority before the maternal mortality related to abortion was reduced’.

## Discussion

Our findings reveal that despite political will and high priority to abortion services at the federal level, there remain considerable challenges in maintaining priority and hence implementing abortion services at regional and local levels. These challenges seem to be exacerbated by the lack of funding and the inadequacy of indicators or reporting systems that ensure accountability and that provide grounds for giving priority to abortion among the series of health programmes and priorities that local health authorities are set to implement. Finally, the situation is further challenged by the contested nature of the abortion issue itself, both in the general population, but also among health bureaucrats and hospital leaders. This study casts light on the complex and entangled processes of turning national-level priorities into on-the-ground practice. In what follows, we will address each of these challenges and discuss their possible implications for a future framework for understanding the gap between national-level priority setting and on-the-ground services implementation.

When a health service is set as a priority it may be assumed that financial support to implement the service will follow. Yet our study finds that this was not necessarily the case for abortion services in Ethiopia. Similarly, Essue and Kapiriri (2020) have shown that in Uganda the lack of financial and human resources was among the main challenges to a successful priority-setting process and implementation of stated priorities. Concern about financial constraints was also seen as a main worry to fair and legitimate priority-setting processes as expressed by health bureaucrats in another study from Ethiopia (Petricca and Bekele, 2018). The lack of funds created a feeling among key stakeholders that the prioritization process was futile and hence hampered the institutionalization and improvement of the national priority-setting processes (Essue and Kapiriri, 2020). The lack of funding was at the time of the study further challenged by the Covid-19 pandemic and internal conflict in the Northern regions of Ethiopia, which shifted funding away from health services (Feyssa and Gebru, 2022).

Bridgman-Packer and Kidanemariam (2018) have documented that the willingness of the Ethiopian government to change the abortion law was an important factor behind the implementation of safe abortion services across the country. Our study finds that this political will is not sufficient to improve access and quality of services. There seemed to be a dissonance between federal-level priorities and the reality of translating these to feasible priorities and service implementation on regional, local and eventually clinical levels. Hipgrave *et al*. (2014) have pointed to the same dissonance in their research on priority setting at the regional and local health administration level in low- and middle-income countries (LMICs). They state that ‘[the] problem remains that many donors and national authorities are expecting meso-level [regional level] decision-makers to introduce interventions or fund activities before it is feasible to prioritize them’. What they postulate is that priority-setting processes in LMICs often are disconnected from the reality on the ground, arguing that context in terms of a functioning healthcare system, local politics, availability of data, ownership and vested interest play more important roles in the actual priority setting and subsequent implementation than the priority setting criteria themselves (Hipgrave *et al*., 2014). If this is not considered, the actual implementation of the set priority will prove difficult. As seen in our study, several regional health bureaucrats similarly felt that it was not feasible to fully implement abortion as a priority because federal authorities were not providing them with the necessary resources, neither financially nor in terms of human resources. With numerous other health services in need of funding, it seemed difficult to make funds for abortion services a main priority.

In the case of Ethiopia, it is important to reflect upon how the Covid-19 pandemic and the internal conflict in the Northern regions affected health priorities. It has been reported that there was an increased shortage of commodities for abortion during the initial phase of the pandemic as resources were redirected towards the pandemic (The African Population and Health Research Center, IPAS Africa Alliance, Amref Health Africa *et al*., 2021). Women faced challenges in accessing abortion care as movement restrictions made it difficult to leave the house (The African Population and Health Research Center, IPAS Africa Alliance, Amref Health Africa *et al*., 2021; Feyssa and Gebru, 2022). The conflict left thousands of people internally displaced and damaged vital healthcare infrastructure (Center for Preventive Action, 2023). A considerable escalation in sexual violence during the conflict, combined with a lack of access to essential sexual and reproductive health services was also reported (Mishori *et al*., 2023). One report found that 27% of the victims of conflict-related sexual violence had an unwanted pregnancy (Mishori *et al*., 2023). In such circumstances, access to safe abortion becomes even more urgent and should be a high priority. The study participants did acknowledge that the conflict and war had made it more difficult to find funding for abortion services, especially since several donors withdrew their support due to the conflict. This is an important context to keep in mind in the interpretation of this study’s result.

In our study, we also found that abortion services were not adequately monitored and evaluated. Indicators and data on abortion were according to several study participants insufficient or missing as compared with monitoring and evaluation of other health services. Although a national registry of abortion exists in Ethiopia, not all our study participants were aware of this nor trusted the data. The registry is not open to the public and the only indicator it measures is the number of women that received abortion care at public clinics. Inadequate indicators and monitoring of abortion services were also found in Bridgman-Packer and Kidanemariam’s study from Ethiopia in 2018. Adams (2016) argues that what we count in health is political, suggesting that politics shapes which health services are being reported on and hence accounted for. Without accountability, it is more likely that ownership and responsibility towards further prioritizing and implementing the set health service is diluted. This could help explain the weakening of priority to abortion as the policy travelled down the healthcare system in Ethiopia. With regional health authorities being held more accountable for other health services like HIV treatment and deliveries, it is fair to assume that abortion did not receive the same attention as other reproductive health services, despite being listed as a high-priority service by the federal government. For abortion to be fully prioritized at all levels of the health system, it is important to have indicators that also show the extent to which the service is being utilized, the quality of care and actual availability and accessibility of abortion services. Such indicators would not only hold bureaucrats accountable for abortion services but also function as a reminder of the political will to provide such services, making it easier for regional and local bureaucrats to implement a somewhat disputed service.

In a study from Senegal, health workers were found to record suspected induced abortions as miscarriages to mask the actual numbers of induced abortions conducted, rewriting the narrative about the type of abortions happening in the public health system (Suh, 2018). Suh (2018) describes how such reporting practices ‘reproduce(s) the invisibility of induced abortion within the health system by measuring hospitals’ capacity to treat complications of unspecified abortions’. This illustrates the importance of data in shaping the story told about abortion, a story which can have substantial consequences for how important the abortion issue is perceived to be and hence to what extent it is prioritized. As pointed out by a participant in our study, the narrative in Ethiopia about the successful reduction in deaths due to a reduction in unsafe abortion is used to undermine the importance of continuing to keep abortion as a priority. Abortion was no longer seen as an important priority because the deaths due to abortion were allegedly reduced or rather were not as visible. The crucial role that reporting, data and subsequent narrative construction play in policy formation is easily underestimated. Glassman *et al*. (2012) and Hipgrave *et al*. (2014) have shown how the lack of appropriate data and insufficient or biased monitoring and evaluation are hampering fair priority-setting processes. Other studies have also shown that lack of data is a hindrance to informed priority setting and leads to more informal processes where anecdotal evidence and personal opinions are used as evidence for what should be prioritized (Kapiriri and Martin, 2006; Gordon *et al*., 2009; Barasa *et al*., 2017).

Since our findings suggest that a moral stand against abortion is strongly present across the bureaucracy line, it likely reduced the likelihood of seeing the service prioritized. This caused an additional obstacle in translating national priorities to regional- and local-level services. Similar roles of attitudes, values and stigma in providing and accessing abortion services have been well described in the literature (Kumar *et al*., 2009; Kumar, 2013; 2018; Shellenberg *et al*., 2014; Coast and Murray, 2016; De Zordo, 2018). We argue that also in an Ethiopian context, morally condemning values against abortion affect how the service is being prioritized and implemented.

What we find in our study is that health bureaucrats play an important role in granting or hindering access to abortion services as they control the true priority of the services in terms of accountability towards the service and the resources being made available for implementation. The values of hospital managers, regional and federal health bureaucrats are, according to our findings, likely shaping the degree of attention and hence priority given to abortion services. As stated by one of the study participants ‘(…) despite the presence of this very good legislation, the officials, even at the ministry level, at all levels of the system, are quite restrictive’. In Haaland *et al*.’s (2020) study of health bureaucrats and their navigation of abortion policy in rural Zambia, it is shown how lower-level health bureaucrats play a crucial role in implementing safe abortion services. While the study concludes that lack of political will and unclear guidance from central authorities is what hinders local health bureaucrats from implementing safe abortion services, our study suggests that the dynamics are different in Ethiopia. Our material similarly indicates that there are a few key individuals who have pushed this agenda forward, but the policy does not seem to be reflected in the general sentient even at the federal level.

Thus, this study suggests that the values that federal and regional health bureaucrats and hospital managers hold and the local political environment in which they operate play an important role in shaping agreed-upon national priorities. Similarly, a study from Uganda found that local health bureaucrats aligned their priorities more towards local political agendas than national ones (Essue and Kapiriri, 2020). In consequence, funds were spent on other health services than the ones that had been set as a national priority (Essue and Kapiriri, 2020). In the priority-setting literature, the decisive role of context in real-life priority decisions has been acknowledged. Contextual dynamics like health care infrastructure, political agendas and the influence of lobbying groups are some of the factors affecting priority decisions (Hipgrave *et al*., 2014).

Considering the political nature of allocating budgets, it is pertinent to look at what happens after decisions are made to prioritize contested and controversial services, such as abortion. According to Tadele *et al*. (2019), abortion has been a silenced topic in Ethiopia, kept outside major public debate not to cause negative reactions to the current law. When a topic is silenced, information about it does not necessarily reach the people who need it. Many health bureaucrats in Ethiopia still do not have sufficient knowledge about the consequences of unsafe abortions and the importance of safe abortion services to reduce maternal death and morbidity. Some also have a moral stance against abortion and are hence reluctant to implement abortion services even though it is a stated priority. This may explain why budgets are not being allocated for abortion services, rooms for abortion are reallocated for other purposes and health workers trained in safe abortion care are being placed in hospital departments where abortions are not performed or leave their work on their own initiative.

By silencing the abortion issue, the legitimacy of making abortion services a priority is challenged. Silence undermines transparency which is one of the widely agreed-upon principles for a fair priority-setting process (Daniels and Sabin, 2008). Yet, by publicly disclosing and discussing safe abortion, one may risk that abortion services lose legitimacy as they may not be publicly endorsed and hence would not be prioritized. Looking at the priority of safe abortion services thus becomes an interesting lens into looking at the implications of priority setting in health. The case of the Ethiopian abortion policy raises the complex question of whether and how a health service should be prioritized if the majority is morally against it. This study thus calls for scholars to examine questions such as how to practically deal with values and politics in the priority-setting processes. There is also a need to further explore how incentives such as indicators shape the implementation of prioritized health care.

## Conclusion

We believe that our study can help shed light on the complexity of priority setting in practice. Through looking at a contested health issue like abortion services, we have highlighted real-life challenges of setting and implementing health priorities. Our study demonstrates that we need a more in-depth understanding of how priorities are experienced, interpreted and executed at various levels of the health system, and how the concrete priorities are constantly negotiated and renegotiated at different systemic levels until they eventually turn into an actual health service offered to the population or gradually vanish as if the challenge did not exist.

## Data Availability

The data underlying this article cannot be shared publicly for the privacy of individuals that participated in the study. The data will be shared on reasonable request to the corresponding author.

## Reflexivity statement

This is a qualitative study and care has been taken to be conscious about how the background of the authors has shaped the study. Data collection, interpretations and drafts have been discussed in several rounds to uncover bias and be true to the material. One author is Ethiopian. She is based in Ethiopia, is a trained nurse and PhD. She has extensive experience in conducting qualitative research and has worked with health policy. The first author is a Norwegian-Jamaican medical doctor and PhD-student; she has spent time in Ethiopia during data collection and this study is part of her ongoing PhD project on abortion in Ethiopia. The last three authors are Norwegian, two of which are professors in respectively medical ethics and anthropology. They have spent considerable time in Ethiopia before and conducted several research projects in the country. The last author is a post-doc with in-depth knowledge of abortion policy in Zambia having conducted her PhD on this topic. The authors have a mix of seniority and expertise which have brought different theoretical perspectives to the study expanding the understanding of the findings. All have experience with qualitative research. Though the majority of the authors are Norwegian, several have conducted research in Ethiopian before, and conscious effort has been made to be sensitive to the Ethiopian context and listen to advice from the Ethiopian author and partners in Ethiopia.

## References

[R1] Adams V . 2016. Metrics of the Global Sovereign: numbers and Stories in Global Health. In: Adams Vincanne (ed). *Metrics, what counts in global health*, 1st edn. Durham, London: Duke University Press, 19–54.

[R2] The African Population and Health Research Center, IPAS Africa Alliance, Amref Health Africa et al. 2021. Impact of the COVID-19 pandemic on sexual and reproductive health services in Burkina Faso, Ethiopia, Kenya, Malawi and Uganda. Report.

[R3] Alebachew A, Yusuf Y, Mann C, Berman P; FMOH. 2015. Ethiopia’s Progress in Health Financing and the Contribution of the 1998 Health Care and Financing Strategy in Ethiopia. Harvard T.H. Chan School of Public Health; Breakthrough International Consultancy, PLC; and Ethiopian Federal Ministry of Health. Boston, Massachusetts and Addis Ababa, Ethiopia.

[R4] Attride-Stirling J . 2001. Thematic networks: an analytic tool for qualitative research. *Qualitative Research* 1: 385–405.

[R5] Barasa EW, Cleary S, Molyneux S, English M. 2017. Setting healthcare priorities: a description and evaluation of the budgeting and planning process in county hospitals in Kenya. *Health Policy & Planning* 32: 329–37. 27679522 10.1093/heapol/czw132 PMC5362066

[R6] Bercu C, Jacobson LE, Gebrehanna E et al. 2022. “I was afraid they will be judging me and even deny me the service”: experiences of denial and dissuasion during abortion care in Ethiopia. *Frontiers in Global Women’s Health* 3: 984386. 10.3389/fgwh.2022.984386 PMC9663468 36386432

[R7] Blystad A, Haukanes H, Tadele G et al. 2019. The access paradox: abortion law, policy and practice in Ethiopia, Tanzania and Zambia. *International Journal for Equity in Health* 18: 126. 10.1186/s12939-019-1024-0 PMC6764131 31558147

[R8] Bridgman-Packer D, Kidanemariam S. 2018. The implementation of safe abortion services in Ethiopia. *International Journal of Gynecology & Obstetrics* 143: 19–24. 10.1002/ijgo.12673 30374983

[R9] Center for Preventive Action . 2023. Conflict in Ethiopia. Global Conflict Tracker. https://cfr.org/global-conflict-tracker/conflict/conflict-ethiopia, accessed 20 August 2023.

[R10] Center for Reproductive Rights . 2024. World abortion laws. https://reproductiverights.org/maps/worlds-abortion-laws/, accessed 25 January 2024.

[R11] Coast E, Murray SF. 2016. “These things are dangerous”: understanding induced abortion trajectories in urban Zambia. *Social Science & Medicine* 153: 201–9. 26921835 10.1016/j.socscimed.2016.02.025

[R12] Cromwell I, Peacock SJ, Mitton C. 2015. ‘Real-world’ health care priority setting using explicit decision criteria: a systematic review of the literature. *BMC Health Services Research*. 15: 164. 10.1186/s12913-015-0814-3 PMC4433097 25927636

[R13] Daniels N, Sabin JE. 2008. Accountability for reasonableness: an update. *Bmj* 337: a1850. 10.1136/bmj.a1850 18845595

[R14] De Zordo S . 2018. From women’s ‘irresponsibility’ to foetal ‘patienthood’: obstetricians-gynaecologists’ perspectives on abortion and its stigmatisation in Italy and Cataluña. *Global Public Health* 13: 711–23. 28278744 10.1080/17441692.2017.1293707

[R15] Dibaba Y, Dijkerman S, Fetters T et al. 2017. A decade of progress providing safe abortion services in Ethiopia: results of national assessments in 2008 and 2014. *BMC Pregnancy & Childbirth* 17: 76. 10.1186/s12884-017-1266-z PMC5336611 28257646

[R16] Eregata GT, Hailu A, Geletu ZA et al. 2020. Revision of the Ethiopian essential health service package: an explication of the process and methods used. *Health Systems & Reform* 6: e1829313. 10.1080/23288604.2020.1829313 33300838

[R17] Essue BM, Kapiriri L. 2020. Priority setting for health system strengthening in low income countries. A qualitative case study illustrating the complexities. *Health Systems* 10: 222–37. 34377445 10.1080/20476965.2020.1758596 PMC8330724

[R18] FDRE . 2005. The criminal code of the Federal Democratic Republic of Ethiopia. Proclamation No. 414/2004.

[R19] FDRE, FMoH . 2014. Technical and Procedural Guidelines for Safe Abortion Services in Ethiopia.

[R20] Feyssa MD, Gebru SK. 2022. Liberalizing abortion to reduce maternal mortality: expanding access to all Ethiopians. *Reproductive Health* 19: 151. 10.1186/s12978-022-01457-z PMC9237962 35761348

[R21] FMoH . 2015. Health Sector Transformation Plan II 2020/21-2024/25. *Federal Ministry of Health Ethiopia*, February 2021.

[R22] FMoH Ethiopia . 2019. Essential Health Services Package of Ethiopia.

[R23] Gebrehiwot Y, Fetters T, Gebreselassie H et al. 2016. Changes in morbidity and abortion care in Ethiopia after legal reform: national results from 2008 and 2014. *International Perspectives on Sexual and Reproductive Health* 42: 121–30. 28825903 10.1363/42e1916 PMC5568644

[R24] Gebremariam EA Kidanemariam MB . 2023. An introduction to Ethiopian legal culture. In: Koch S, Kjølstad MM (eds.). *Handbook on Legal Cultures: A Selection of the World’s Legal Cultures*. Cham: Springer International Publishing, 419–62.

[R25] Geleto A, Markos J. 2015. Awareness of female students attending higher educational institutions toward legalization of safe abortion and associated factors, Harari Region, Eastern Ethiopia: a cross sectional study. *Reproductive Health* 12: 19. 10.1186/s12978-015-0006-y PMC4383196 25880854

[R26] Glassman A, Chalkidou K, Giedion U et al. 2012. Priority-setting institutions in health: recommendations from a center for global development working group. *Global Heart* 7: 13–34. 25691165 10.1016/j.gheart.2012.01.007

[R27] Gordon H, Kapiriri L, Martin DK. 2009. Priority setting in an acute care hospital in Argentina: a qualitative case study. *Acta Bioethica* 15: 184–92.

[R28] Haaland MES, Haukanes H, Zulu JM, Moland KM, Blystad A. 2020. Silent politics and unknown numbers: rural health bureaucrats and Zambian abortion policy. *Social Science & Medicine (1982)* 251: 112909. 10.1016/j.socscimed.2020.112909 32179365

[R29] Ham C . 1997. Priority setting in health care: learning from international experience. *Health Policy* 42: 49–66. 10173493 10.1016/s0168-8510(97)00054-7

[R30] Harris D, Baird S, Ford K et al. 2021. The Impact of COVID-19 in Ethiopia: Policy Brief. Oxford Policy Management.

[R31] Hellerstein E . 2023. Roe’s repeal has energized Africa’s anti-abortion movement. Coda Story. https://www.codastory.com/waronscience/dobbs-abortion-global-impact/, accsessed 12 May 2024.

[R32] Hipgrave DB, Alderman KB, Anderson I, Soto EJ. 2014. Health sector priority setting at meso-level in lower and middle income countries: lessons learned, available options and suggested steps. *Social Science & Medicine* 102: 190–200. 24565157 10.1016/j.socscimed.2013.11.056

[R33] Holcombe SJ, Gebru SK. 2022. Agenda setting and socially contentious policies: Ethiopia’s 2005 reform of its law on abortion. *Reproductive Health* 19: 218. 10.1186/s12978-021-01255-z PMC9195348 35698196

[R34] Institute for Health Metrics and Evaluation . 2023. Ethiopia. https://www.healthdata.org/ethiopia, accessed 27 June 2023.

[R35] Kapiriri L, Martin DK. 2006. Priority setting in developing countries health care institutions: the case of a Ugandan hospital. *BMC Health Services Research* 6: 127. 10.1186/1472-6963-6-127 PMC1609114 17026761

[R36] Kapiriri L, Razavi D. 2017. How have systematic priority setting approaches influenced policy making? A synthesis of the current literature. *Health Policy* 121: 937–46. 28734682 10.1016/j.healthpol.2017.07.003

[R37] Kumar A . 2013. Everything is not abortion stigma. *Women’s Health Issues* 23: 329–31. 10.1016/j.whi.2013.09.001 24183406

[R38] Kumar A . 2018. Disgust, stigma, and the politics of abortion. *Feminism & Psychology* 28: 530–8. 30542236 10.1177/0959353518765572 PMC6236644

[R39] Kumar A, Hessini L, Mitchell EMH. 2009. Conceptualising abortion stigma. *Culture, Health & Sexuality* 11: 625–39. 10.1080/13691050902842741 19437175

[R40] McLean E, Desalegn DN, Blystad A, Miljeteig I. 2019. When the law makes doors slightly open: ethical dilemmas among abortion service providers in Addis Ababa, Ethiopia. *BMC Medical Ethics* 20: 60. 10.1186/s12910-019-0396-4 PMC6727528 31488124

[R41] Micah AE, Bhangdia K, Cogswell IE et al. 2023. Global investments in pandemic preparedness and COVID-19: development assistance and domestic spending on health between 1990 and 2026. *The Lancet Global Health* 11: e385–413. 36706770 10.1016/S2214-109X(23)00007-4 PMC9998276

[R42] Mishori R, McHale T, Green L, Olson RM, Shah PK. 2023. Conflict-related sexual violence continues in Tigray, Ethiopia. *The Lancet* 402: 1023–5. 10.1016/S0140-6736(23)01734-8 37634524

[R43] Moore AM, Gebrehiwot Y, Fetters T et al. 2016. The estimated incidence of induced abortion in Ethiopia, 2014: changes in the provision of services since 2008. *International Perspectives on Sexual and Reproductive Health* 42: 111–20. 28825902 10.1363/42e1816 PMC5568682

[R44] Partnership and Cooperation Directorate, FMoH . 2022. Ethiopia National Health Accounts Report 2019/20.

[R45] Petricca K, Bekele A. 2018. Conceptualizations of fairness and legitimacy in the context of Ethiopian health priority setting: reflections on the applicability of accountability for reasonableness. *Developing World Bioethics* 18: 357–64. 28544136 10.1111/dewb.12153

[R46] QSR International Pty Ltd . 2020. NVivo software (released in March 2020).

[R47] Seixas BV, Dionne F, Mitton C. 2021. Practices of decision making in priority setting and resource allocation: a scoping review and narrative synthesis of existing frameworks. *Health Economics Review* 11: 2. 10.1186/s13561-020-00300-0 PMC7789400 33411161

[R48] Sheehy G, Dozier JL, Mickler AK et al.. 2021. Regional and residential disparities in knowledge of abortion legality and availability of facility-based abortion services in Ethiopia. *Contraception: X* 3: 100066. 10.1016/j.conx.2021.100066 PMC8267565 34278291

[R49] Shellenberg KM, Hessini L, Levandowski BA. 2014. Developing a scale to measure stigmatizing attitudes and beliefs about women who have abortions: results from Ghana and Zambia. *Women & Health* 54: 599–616. 25074064 10.1080/03630242.2014.919982

[R50] Suh S . 2018. Accounting for abortion: accomplishing transnational reproductive governance through post-abortion care in Senegal. *Global Public Health* 13: 662–79. 28287330 10.1080/17441692.2017.1301513 PMC6129976

[R51] Suh S . 2020. What post-abortion care indicators don’t measure: global abortion politics and obstetric practice in Senegal. *Social Science & Medicine* 254: 112248. 10.1016/j.socscimed.2019.03.044 PMC6776722 31029482

[R52] Tadele G, Haukanes H, Blystad A, Moland KM. 2019. ‘An uneasy compromise’: strategies and dilemmas in realizing a permissive abortion law in Ethiopia. *International Journal for Equity in Health* 18: 138. 10.1186/s12939-019-1017-z PMC6764138 31558166

[R53] Wada T . 2008. Abortion law in Ethiopia: a comparative perspective. *Mizan Law Review* 2: 1–32.

[R54] World Bank Group . 2016. Ethiopia public expenditure review: World Bank, Washington, DC, 26. http://hdl.handle.net/10986/24370, accessed 04 January 2024.

[R55] World Health Organization . 2024. First case of COVID-19 confirmed in Ethiopia. WHO | Regional Office for Africa. https://www.afro.who.int/news/first-case-covid-19-confirmed-ethiopia, accessed 03 May 2024.

[R56] Yeshambel Wassie A, Berhe Lemlem S, Boka A. 2021. Knowledge, practice and associated factors towards medication abortion among reproductive-age women in sexual and reproductive health clinics of Addis Ababa, Ethiopia, 2018: cross-sectional study. *International Journal of Women’s Health* 13: 489–99. 10.2147/IJWH.S297626 PMC8163624 34079386

[R57] Zimmerman LA, Karp C, Kassa M et al. 2022. What is the relationship between contraceptive services and knowledge of abortion availability and legality? Evidence from a national sample of women and facilities in Ethiopia. *Health Policy & Planning* czac103. 10.1093/heapol/czac103 PMC10019562 36440697

